# A diabetic foot wound healing assessment tool: A scoping review

**DOI:** 10.1016/j.heliyon.2023.e15736

**Published:** 2023-04-25

**Authors:** Suriadi Jais, Kharisma Pratama

**Affiliations:** Department Medical – Surgical Nursing, Post Graduate School of Nursing, Institute of Technology and Health,Muhammadiyah Pontianak, Indonesia

**Keywords:** Diabetic ulcer, Assessment, Reliability, Validity, Wound healing

## Abstract

**Background:**

Indonesia faces a challenge in controlling the burden of diabetic foot ulcers, which necessitates a nursing care management approach to optimize the healing of complications by accurately monitoring wound healing progress using wound assessment tools.

**Methods:**

This literature review, which is part of a scoping study framework, searched electronic databases including PubMed, ScienceDirect, EBSCOhost, and Google Scholar to find papers relevant to the Indonesian context. Five papers were chosen from a total of 463 papers discovered.

**Results:**

The diabetic foot ulcer wound assessment tools DFUAS (diabetic foot ulcer assessment scale), DMIST (deep, maceration, infection, size, and tunneling), and MUNGS (maceration, undermining, necrotic, granulation, and symptoms/signs) were identified in the literature review. For leg ulcers, LUMT (leg ulcer measurement tool) and RESVECH 2.0 (Results Expected from Chronic Wound Healing Assessment) were used. DMIST, DFUAS, and MUNGS are used to predict healed and non-healing wounds. LUMT determines the evaluation and documentation of leg ulcers, and RESVECH 2.0 is designed to shorten the duration of chronic wound occurrence. The psychometric properties of the DMIST scale were identified, including reliability, validity, and responsiveness.

**Conclusions:**

Five tools for assessing chronic wounds were identified. The predictive validity and responsiveness of the DMIST tool were supported by a sufficient rating based on evidence quality. This scoping review provides an overview of the measurement properties of available assessment tools for diabetic foot ulcers.

## Teaser

Standard practices for the management of DFU include surgical debridement, wound dressings to maintain a moist environment, the control of exudate production, wound off-loading, vascular and infection assessments, and glycaemic control [45]. Comprehensive wound care is important to ensure that wounds heal quickly and do not result in complications such as infections and amputations. Therefore, an accurate wound healing assessment tool is needed to help decrease the prevalence of infections and amputations. As wound healing is a complex process, a variety of factors, such as the size and depth of the wound, the condition and type of granulation, the presence of infection, maceration, necrotic tissue, and exudate production as well as the condition of the edges of the wound, must be taken into consideration when monitoring the healing of a wound [46,27]. These important observable factors can be included in an assessment tool to simplify wound healing evaluations. However, the wound assessment tool must be tested for accuracy prior to clinical use. It must also be deemed reliable and valid for its results to be considered as credible [30] as well for it to be readily available and used in a routine and well-documented manner.

## 1 Introduction

Diabetes mellitus affects approximately 425 million people worldwide and has an annual incidence rate of 6.7–7 per 1000 people in developed countries [1]. Diabetic patients have a 15–25% lifetime risk of developing diabetic foot ulcers (DFU) [2,3], and incidence rates of 37.9–57.5% for the recurrence of DFU [4]. A provincial Indonesian study reported a DFU incidence rate of 66.7% [5]. Similar studies have reported incidence rates of 43–54.3% for the recurrence of diabetic ulcers [6,7].

Wound assessment is critical for determining whether a wound is improving or getting worse. As such, many DFU wound assessment tools have been developed. The leg ulcer measurement tool (LUMT), which originally contained 17 items, has a score range of 0–68, with zero indicating that the wound has completely healed [8,9]. Meanwhile, the DFU assessment scale (DFUAS) that was proposed in 2016 contains 11 domains and has a score range of 0–98, with the higher scores indicating higher wound severity [7]. The maceration, undermining, necrotic, granulation, and symptoms (MUNGS) tool that was developed in 2016 consists of five items and has a score range of 0–15, with zero indicating that the ulcer has completely healed [10]. Similarly, the depth, maceration, inflammation/infection, size, tissue type of the wound bed, type of wound edge, and tunnelling/undermining (DMIST) tool that was developed in 2019 consists of seven items and has a score range of 0–34, with zero indicating that the ulcer has completely healed [11]. The depth, extent of bacterial colonisation, phase of healing and associated aetiology (DEPA) tool was developed to predict the likelihood of foot amputation in diabetic patients. Each item is scored on a scale of 1–3, with a total score of 3–12 [12]. The original photographic wound assessment tool (PWAT) was first developed in 2000 and validated for the assessment of chronic pressure ulcers and leg ulcers. However, the revised PWAT (revPWAT) now consists of eight domains and has a score range of 0–32, with zero representing a completely healed wound [13]. The curative health services (CHS) tool was developed in 2003 and comprises six grades that examine wound depth as well as the presence of abscesses, osteomyelitis, and necrotic tissue. The tool was validated by using it thrice to predict the healing of neuropathic DFU at the 20th week of care at all CHS wound care facilities [14]. Meanwhile, both the non-healing, exudate increase, red tissue, debris, smell criteria (NERDS) tool and the size, temperature, osteomyelitis oedema, exudate, smell criteria (STONES) tool were developed in 2006 to identify infected wounds in diabetic patients [15,16]. In 2016, the International Working Group of the Diabetic Foot (IWGDF) developed the perfusion, extent, depth, infection, and sensation scale (PEDIS) that uses five variables to classify and predict the likelihood of infection [17]. The clinical signs and symptom checklist (CSSC) tool was developed in 2010 and contains 12 items to identify infected diabetic wounds [18]. Similarly, the diabetic foot infection wound score (DFIWS) tool was created in 2009 and includes a 10-item wound score that ranges from 3 to 49, from the least to the most severe, and a semi-quantitative grading of both wound measurement and various infection parameters [19]. The chronic wound healing assessment tool (RESVECH) consists of 10 items and a score of 0–40 to assess chronic wound healing as well as diabetes, with zero indicating that the wound has completely healed [20].

The abovementioned scales or tools are currently used to classify wounds, identify infected wounds, predict the likelihood of amputation, and monitor wound healing. Therefore, multiple wound assessment tools for diabetic feet have been developed and tested for reliability and validity. However, the accuracy and frequency of their use in clinical practice to assess specific diabetic wounds have not been clearly described. The present study examines the tools that are used to assess wound healing, particularly DFUs. As such, it is essential to examine which tools clearly describe the assessment of wounds for wound care management in diabetic patients. The findings of the present study will provide adequate information for the selection of suitable wound healing assessment tools that support optimal diabetic wound care.

### 1.2 Research questions

The research questions include:

• How reliable and valid are these diabetic ulcer assessment tools?
• How responsive are these wound assessment tools, and how do they affect wound care management in terms of wound healing?

## 2 Research objectives

The objectives of the present review include:

• To summarise the evidence of the accuracy, reliability, and validity of a DFU wound assessment tool; and
• To organise this evidence in clinical settings.

The findings of the present review will benefit the clinical practice of clinicians who care for diabetic ulcer patients.

## 3 Methods

**Fig. 1:**
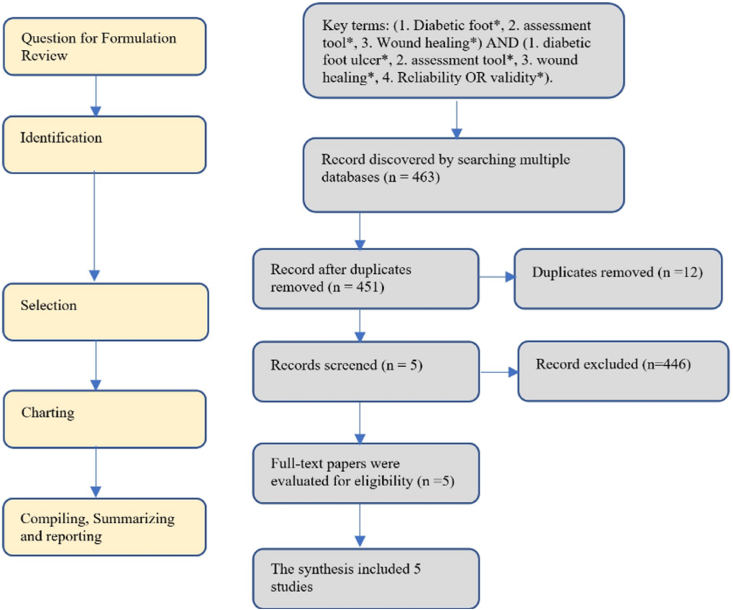
A PRISMA flowchart of the scoping review, which included a total of five articles.

As no review protocols have been published, the preferred reporting items for systematic reviews and meta-analyses extension for scoping reviews (PRISMA-ScR) checklist was used to conduct the current review (Fig. 1). This scoping review was reviewed by the Ethics Committee of the Institute of Technology and Health Muhamamdiyah Pontianak, (no: 280a/II.1.AU/KET.ETIK/IX/2022).

## 4 Eligibility criteria

The article search was restricted to only include original articles and case reports published in English and Indonesian, regardless of the clinical setting, such as inpatient or home care/community, or year of publication. Articles that included DFU participants of all ages who were receiving wound care management with assessment tools were included. Proceedings, conference abstracts, letters to the editor, editorials, guidelines, protocols, literature reviews, and meta-analyses were not included.

## 5 Article sources

In January 2023, a search was conducted on several bibliographic databases, such as PubMed, ScienceDirect, EBSCOhost, and Google Scholar, to compile papers that had been published between 2015 and 2022, with an emphasis on Indonesia and that met the inclusion criteria.

## 6 Article search

Keywords that were based on the research questions and related to diabetic, foot ulcer, assessment tools, reliability, validity, and wound healing were used to search the terms and medical subject headings. The Boolean operator “AND” was then used to combine these keywords. The search strategy was as follows: [1. Diabetic foot*, 2. assessment tool*, 3. wound healing*] AND [1. diabetic foot ulcer*, 2. assessment tool*, 3. wound healing*, 4. reliability OR validity*].

## 7 Article selection

Both authors, SJ and KP, used the Rayyan scoping review tool to independently assess the titles and available abstracts of all the studies identified during the initial search [21]. Articles that met the inclusion criteria but lacked adequate abstract details were included for full-text screening, and were evaluated based on the inclusion criteria. As the current review focused on wound assessment tools for diabetic patients, articles that lacked outcomes for assessment tools and diabetic ulcers in patients with chronic wounds were excluded. Disagreements on article selection were resolved via discussions.

## 8 Data extraction and management

The first author, SJ, summarised the wound assessment tool and diabetic foot findings, while the second author, KP, verified the extracted data. Disagreements on the extracted data were resolved via discussions. The extracted data included the characteristics of the sample such as the total sample size, clinical setting, and human subjects, the characteristics of wound healing, such as wound assessment tools and scores, and comments on the data collection.

## 9 Article source selection

The initial article search yielded 463 articles. After duplicate article were removed (n = 12), the titles and abstracts of the remaining 451 articles were scanned to determine if they met the inclusion criteria. This resulted in the exclusion of 446 articles After the full texts had been read and assessed against the review questions, only five articles were selected for inclusion in the final dataset (Fig. 1).

## 10 Results

The five selected articles, that had been published between 2015 and 2022, were reviewed. All five studies investigated tools for the quantitative assessment of wound healing [7,8,10,11,20]. Only three studies developed wound healing assessment tools for DFUs [7,8,11], while the remaining two studies developed wound healing assessment tools for chronic leg ulcers [10,20]. A review of the studies revealed three major themes, namely, wound healing, assessment tools, and DFUs.

As seen in Table 1, the study of the RESVECH 2.0 tool was the most validated as it involved 281 participants. This was followed by studies on the DMIST (n = 153), LUMT (n = 105), MUNGS (n = 75), and DFUAS (n = 41) tools. The reliability of the LUMT, DMIST, and DFUAS tools was assessed using an intraclass correlation coefficient (ICC) analysis, while the MUNGS tool was assessed using the Kappa (κ) test. The reliability of the RESVECH 2.0 and the LUMT tools was assessed using a combination of the Cronbach's alpha coefficient (α) and κ test. The predictive validity of the DMIST, DFUAS, and MUNGS tools was assessed by determining their sensitivity and specificity. Studies on the RESVECH 2.0 tool and LUMT evaluated their construct validity. The purpose of validating the DMIST, DFUAS, and MUNGS tools was to determine their ability to predict healed and non-healed wounds. Studies on the LUMT examined its ability to evaluate and document leg ulcers while studies on the RESVECH 2.0 tool examined its ability to decrease the duration of chronic wounds. Table 2 summarises the clinimetric qualities of the instruments included, categorising each aspect as positive, intermediate, poor, or no information available. Only the DMIST, DFUAS, and MUNGS had information on responsiveness.

**Table 1:** A summary of the five selected articles.

Author(s)	Aim	Design	Participants and Samples	Themes	Reliability and Validity		Wound Healing Assessment	Conclusion
					Reliability	Validity		
Defa Arisandi et al. (2016)	To evaluate the validity, construct validity, and predictive validity of DFUAS tool.	Prospective cohort study.	70 type 2 diabetes patients with diabetic wounds, of which 41 were included in the predictive validity analysis.	Assessment tool, wound healing, and DFUs.	Total Bates-Jensen wound assessment tool (BWAT) score and pressure ulcer scale for healing (PUSH) score. Agreement with gold standard and wound surface area. Intraclass correlation coefficient (ICC) total of DFUAS = 0.98, r = 0.83 to 0.92.	Sen: 98%, Spe: 71%.	Predicted healing and non-healing DFUs in 4 weeks.	DFUAS is a valid tool for assessing DFUs.
Makoto Oe et al. (2020)	To evaluate the validity of the DMIST tool.	Secondary analysis: Prospective cohort study and randomised controlled trial (RCT).	153 type 2 diabetes patients with diabetic wounds.	Assessment tool, wound healing, and DFUs.	Total ICC of the DMIST tool was 0.905.	Sen: 86%, Spe: 79%.	Predicted healing and nonhealing DFUs in 4 weeks.	The DMIST tool contributes to the evidence for diabetic foot management.
Suriadi (2019)	To determine the critical cut-off point of the MUNGS tool to classify wound healing.	Observation study.	75 patients.	Assessment tool, wound healing, and DFUs.	Kappa (κ): Almost perfect.	Sen: 84.91%, Spe: 81.82%.	Predicted healing and nonhealing DFUs.	The MUNGS tool was very accurate in determining the average score.
Isabelle Andrade Silveira et al. (2022)	To validate the construct and reliability of the Portuguese version of the LUMT.	Methodological research based on the theoretical-methodological concepts of psychometry.	105 participants.	Measurement tool and leg ulcers.	Cronbach's Alpha (α) = 0.711, ICC = 0.823.	Construct validity: The proposed adapted LUMT had 12 items and produced a score that varied by 0 to 48 from the original 0 to 68.	Evaluated leg ulcers.	The adapted LUMT scale is an important tool in the evaluation and documentation of leg ulcers.
Alexandre Marques Rodrigues et al. (2022)	To conduct a cross-cultural adaptation and psychometric analysis of the Portuguese version of the RESVECH 2.0.	Quantitative and correlational study.	281 participants.	Validation, chronic wounds, and assessment.	α = 0.73, κ = 0.519–0.975.	Construct validity: Kaiser-Meyer-Olkin (KMO) = 0.776, considered average/good. Bartlett's sphericity measure = < 0.001.	To shorten the duration of chronic wounds.	European–Portuguese version of the RESVECH 2.0 was acceptable with internal consistency and good validity.

**Table 2:** Clinimetric evaluation of measurement tools.

Terwee checklist [22] (score: + = positive; ? = intermediate; – = poor; 0 = no information available)									
Instrument tool	Content validity	Internal consistency	Criterion validity	Construct validity	Reproducibility		Responsiveness	Floor or ceiling effect	Interpretability
					Agreement	Reliability			
DFUAS	+	0	+	+	0	+	+	0	0
DMIST	+	0	+	+	0	+	+	0	?
MUNGS	+	0	+	0	0	+	+	0	0
RESVECH 2.0.	+	+	+	+	?	+	0	0	?
LUMT	+	+	0	0	?	+	0	0	0

## 12 Discussion

To date, 13 wound assessment tools have been developed for diabetic patients. The current review was limited to articles published between 2015 and 2022 that examined tools for the assessment of wound healing in diabetic patients. It is also the first review to describe the reliability and validity of wound healing assessment tools for DFUs. Only five articles that examined diabetic foot wound assessment tools were deemed to be relevant for the purposes of this current review as they had been tested for reliability and validity. These tools also assess and monitor patient conditions using a host of psychometric characteristics [23]. Although one study into the DFUAS tool determined that it had good validity, and also examined its sensitivity and specificity, a normality test should have been conducted as the sample of the study was small [7]. Another study examined the inter-rater reliability (IRR) of the DFUAS tool. However, the results were deemed low quality as photographs were used to demonstrate the use of the DFUAS tool [24]. This is because, although digital photography is a non-invasive, quantitative, fast, and cost-effective method of assessing wound healing, it cannot be used to thoroughly assess wound tissue damage or fully describe underlying tissue damage [25]. Furthermore, as wound images must be captured in a controlled environment, it is impractical to do so in everyday clinical practice as the colour analysis and wound dimension measurements would suffer high rates of error and produce highly erroneous results. As such, the reliability and validity of the DFUAS tool warrants a re-evaluation.

A secondary analysis of the data produced by Arisandi et al. (2016) was used to develop a new seven-domain DFU assessment scale called the DMIST tool [7], which has been found to be a reliable and valid assessment tool for DFUs [11]. Three of the seven items on the DMIST scale, namely, depth, size, and maceration, have been found to be significantly correlated to wound healing, while a comparison indicated that the most frequently occurring components were wound extension size, depth, and exudate production or maceration [26], which are important indicators when monitoring wound healing [27,28,29]. In wound care management, a total score scale is essential for assessing wound healing [30,31,32]. For instance, a lower number or zero indicates that the wound has completely healed and vice versa, while changes in the total scale score of a wound assessment are used to evaluate the scale parameters. However, although the total score scale of the DMIST tool is able to explain the severity of diabetic wounds, it differs significantly with the Wagner wound classification (Grade I vs. Grade II/III vs. Grade IV/V) [11].

Therefore, as the DMIST tool is currently being used in Indonesia and Japan to assess diabetic wound progression in a clinical setting, it should be re-evaluated using primary data from studies that have large sample sizes and the 12-week recommended healing time for chronic wounds [33]. Another predictive validity study similarly determined that nine was the cut-off score for the DMIST tool. The DMIST tool was also found to have a good balance between sensitivity and specificity, and was able to successfully predict which diabetic wounds would and would not heal within 12 weeks [33]. However, the quality of the results warrants improvement.

The MUNGS tool is another wound healing assessment tool that examines psychometric characteristics. The IRR of the MUNGS tool was almost excellent (0.81) among wound care nurses, and substantial (0.69) among nursing students [8]. It also had a good balance of sensitivity and specificity. However, although the scale of the MUNGS tool was reliable and valid, the sample of the validity test was small and the recommended properties of the psychometric tests should be improved [34]. Therefore, the scale of the MUNGS tool warrants re-evaluation with a larger sample size.

The LUMT wound assessment tool for leg and diabetic ulcers was developed in Canada in 2004 [9]. A follow-up study confirmed that 12 items could be used in clinical practice in Brazil. However, the “depth” and " bioburden assessment " variables were removed from the factor analysis as they did not vary or correlate with the scale items. In terms of depth, most of the participants had superficial venous ulcers. Therefore, the non-variability of this item was deemed justified. A factor analysis was used to assess the construct validity, while internal consistency and stability were used to assess reliability. However, the results were deemed low quality as a predictive validity investigation was not conducted. Another study attempted to validate the LUMT by assessing its construct validity, concurrent validity, IRR, intra-rater reliability, and sensitivity to change [9]. As a panel of nine wound care specialists agreed that all the relevant domains had been included and all the responses were appropriate, it confirmed the validity of the construct. However, the study failed to explain how a consensus was reached or how biases were minimised. Apart from the LUMT, all the other wound healing assessment instruments lack comprehensive and quality validity assessments. The LUMT scale appears to have adequate IRR and intra-rater reliability, and is sensitive enough to detect monthly changes in ulcer healing. However, the concurrent validity of the LUMT has been called into question as it has not been compared to that of a wound healing measurement tool. Furthermore, it can only detect changes in wound healing but cannot predict wound healing or evaluate wound characteristics [35].

The RESVECH 1.0 tool, which was developed in 2010, is a nine-item tool that assesses physical and psychological components for the measurement and monitoring of the healing of chronic wounds [36]. Experts support its internal consistency. The newer RESVECH 2.0 tool uses a Likert-type scale to measure six dimensions, namely, wound area, depth, edges, type of tissue in the wound bed, exudate production, and signs of infected or inflamed biofilm. The study examined the Portuguese version of the RESVECH 2.0 tool, and determined that it has acceptable internal consistency and validity [20]. However, the psychometric properties of the RESVECH 2.0 tool have not been examined and its results are low quality.

The second research question of this present review aimed to evaluate the responsiveness of the tools and their impact on wound care management in terms of wound healing. Of the five studies that assessed the responsiveness of the MUNG, DFUAS, and DMIST tools that this present review examined, the DMIST tool was the most adequate. According to Makoto Oe et al. (2020), the DMIST scores of subjects in the “wound improved but not healed” group did not change as significantly as those in the “wound healed” group, while that of the “wound static or worsened” group only changed marginally [11]. According to another study, the variables of size, depth, and maceration of the DMIST tool provided the best model of healing. As they play a critical role in the inhibition of wound healing, they are extremely important factors when it comes to assessing wound healing and must be monitored at all times [26]. Another study discovered that good wound healing can be expected if the wound size and DMIST score decrease in the first four weeks [37]. The DMIST tool has a significant impact on wound care management as it is comprised of several factors, namely, wound size, depth, and maceration, which are critical in wound care management and significantly affect wound healing [16,18,19,26,29]. Apart from the depth, size, and maceration of the wound, other items that are important in wound care management, such as the presence of infection, granulation, necrotic tissue in the wound bed, and tunnelling, which promote wound healing as well as the condition of the edges of the wound, must be included and monitored as the total score increases [31,38]. Although the DMIST tool is well known in Indonesia and Japan, the setting and sample size must be taken into consideration in a study so as to improve the results.

The sample sizes of the five studies ranged from 41 to 281 participants, with fewer than 100 participants in the studies on the MUNGS and DFUAS tools [34]. As a sample size of less than 100 is too small to satisfy a normal distribution, these tools have limitations with regard to their use. These studies also used slightly different statistical analysis methods, such as the IRR. In the medical sciences, the κ test and ICC are most commonly used to test an assessment tool [39,40]. There is a general consensus that wound assessment tools should have an ICC or κ that exceeds 0.75–0.80 in clinical research, with 0.90 considered the ideal [41]. Furthermore, the value of α in the studies of the LUMT and RESVECH tools was not standardised, where an α of 0.80 is considered ideal [42]. The five tools used appropriate statistical analysis methods, which appeared to meet the reliability requirements. However, a κ statistical analysis is likely to be recommended for IRR studies [39, 41, 43]. Several methodological issues were discovered while reviewing how these studies validated their instruments. Almost all the studies had a small sample size and used a non-random sampling procedure, which limited the applicability of their findings to other settings and patients or larger populations. Therefore, future studies should comprehensively validate these instruments where gaps exist. Furthermore, larger sample sizes, multi-raters on IRR studies, statistical analyses, and multiple settings should be examined to improve the generalisability of the results. The gold standard in wound care management is the monitoring of wound healing with a reliable and valid wound assessment tool [44]. Therefore, an accurate and tested wound healing assessment tool is required to ensure the quality of health services in wound care management. Although the LUMT and RESVECH tools have been around for a long time, the current review did not find any studies that validated their psychometric properties, such as their ability to predict wound healing. The information provided by wound assessment and monitoring tools must be based on their psychometric properties [44]. Furthermore, these tools have to be reliable and valid to detect response-based changes in wound conditions.

The aim of the current review was to recommend the best tool for the assessment of wound healing as the process of evaluating and monitoring a patient is dependent, at least partly, on selecting the correct tool or scale. However, there is insufficient evidence to confidently recommend any of the existing assessment tools for wound healing. Based on the collected data, the DMIST tool is a promising tool for the monitoring of DFU healing in a clinical setting. However, training is required as the characteristics of the patient, the clinical setting, and the level of experience of the practitioner differ. Furthermore, the tool of choice depends on the conditions in which it is available and established [23]. Similarly, the MUNGS and DFUAS tools can also be used to monitor DFU healing. However, more studies with larger samples and degrees of responsiveness are required to establish its validity. It would also be beneficial to have more clinimetric-based studies to examine the cost-effectiveness of a tool, compare its wound severity to that of other tested wound classifications, and its healing time on the total score scale.

### 12.1 Study limitations

The several limitations of the present review should be taken into consideration when interpreting its findings. Firstly, as linguistic searches were not conducted, tools that were published in languages other than English and Indonesian, such as French, Japanese, Chinese, Spanish, or Italian, were excluded. Furthermore, as this present review only examined studies published between 2015 and 2022, older publications that may contain other tools were excluded. Secondly, although significant efforts were made to synthesise the most frequently examined psychometric characteristics and tools used in clinical settings, some of these studies did not assess the psychometric characteristics.

## 13 Conclusion

Five tools for assessing DFUs were identified. The DMIST, DFUAS, and MUNGS tools solely assess DFUs, while the LUMT and RESVECH 2.0 tools assess leg ulcers. There is evidence of a moderate quality to support the reliability, validity, and responsiveness of the DMIST tool. However, better designed, rigorously conducted, and thoroughly reported studies are needed to validate the limitations of the DMIST tool.

## Author contribution statement

All authors listed have significantly contributed to the development and the writing of this article.

## Data availability statement

Data will be made available on request.

## Conflict of interest statement

The authors declare that they have no conflict of interest.
